# Effects of Dry Needling in Teres Major Muscle in Elite Handball Athletes. A Randomised Controlled Trial

**DOI:** 10.3390/jcm10184260

**Published:** 2021-09-20

**Authors:** Luis Ceballos-Laita, Ricardo Medrano-de-la-Fuente, Elena Estébanez-De-Miguel, Jorge Moreno-Cerviño, María Teresa Mingo-Gómez, Ignacio Hernando-Garijo, Sandra Jiménez-del-Barrio

**Affiliations:** 1 Department of Surgery, Ophthalmology, Otorhinolaryngology and Physiotherapy, Faculty of Health Sciences, University of Valladolid, 42004 Soria, Spain; ricardo.medrano@uva.es (R.M.-d.-l.-F.); jorgemorenocer@gmail.com (J.M.-C.); tmingo@cir.uva.es (M.T.M.-G.); ignacio.hernando@uva.es (I.H.-G.); sandra.jimenez.barrio@uva.es (S.J.-d.-B.); 2 Department of Physiatrist and Nursey, Faculty of Health Sciences, University of Zaragoza, 50010 Zaragoza, Spain; elesteba@unizar.es

**Keywords:** shoulder pain, myofascial pain syndrome, trigger point, dry needling

## Abstract

*Objective*: To determine the effects of dry needling (DN) in active myofascial trigger points in the teres major muscle compared to an untreated control group in pain during throwing actions, shoulder range of motion (ROM), strength, and extensibility of the tissues in professional handball (HB) athletes. *Methods*: A randomised, single-blinded, controlled clinical trial was designed. Thirty HB athletes with shoulder pain were randomly assigned to the DN group (*n* = 15) or control group (*n* = 15). The DN group received a single session of ultrasound-guided DN technique in the teres major muscle. The control group received no intervention. Pain intensity during throwing actions (Numeric Pain Rating Score), shoulder ROM (inclinometer), isometric strength (hand-held dynamometer), and extensibility (inclinometer) were measured before and after treatment. *Results*: DN group showed statistically significant improvements with large effect sizes for pain intensity (*p* < 0.001; E.S: 1.3), internal rotation ROM (*p* < 0.001; E.S: 3.0) and extensibility (*p* < 0.001; E.S: 2.9) compared to the control group. No statistically significant differences were found for isometric strength (*p* > 0.05). *Conclusion*: A single session of DN in the teres major muscle was effective for improving pain intensity during throwing actions, internal rotation ROM and extensibility in HB athletes with shoulder pain.

## 1. Introduction

Handball (HB) is a popular sport, with more than 27 million athletes around the world [1]. This sport involves a wide variety of throwing actions in which the shoulder is exposed to large demands due to repeated overhead motion at high velocity [2,3]. These repeated movements provoke symptoms of overuse injuries. Recent studies have reported that the prevalence of acute or chronic shoulder pain among HB athletes ranges from 36% to 44.2% [4,5].

Previous authors have studied several adaptable risk factors in HB athletes with shoulder pain. Cinematic studies have shown a reduction in the glenohumeral internal rotation range of motion (ROM) and an increment in the external rotation ROM in the throwing shoulder (TS) compared to the contralateral shoulder (non-TS) [6,7,8]. Athletes with glenohumeral internal rotation deficit (GIRD) and external rotation gain (ERG) have presented a higher risk of suffering shoulder pain. The GIRD is currently considered a primary risk factor in the development of shoulder injuries [6,7,9,10,11,12,13,14].

Different authors discussed that these glenohumeral ROM changes occur because of the restriction of the posteroinferior capsule and the posterior band of the inferior glenohumeral ligament [7,10] and because of the stiffness of the posterior shoulder muscles [15]. The restriction of the different tissues of the posteroinferior part of the shoulder shift the humeral head center of rotation to posterosuperior [7,10]. Therefore, the shift of the humeral head generates an attenuation of the anterior part of the capsule and ligaments of the TS, causing different injuries, such as anterior instability, labrum injuries or shoulder impingements [6,7,9,10,11,12,13]. However, a recent study suggested a new paradigm in the shoulder complex [16]. The teres major muscle seems to be an additional component in the glenohumeral stability. This muscle also resists the inferior displacement of the humeral head when the arm is being lifted [16]. So, its dysfunction due to the repetitive movements at high velocity, could be related to glenohumeral ROM changes.

The pain and the changes in glenohumeral ROM in HB athletes could be related to the presence of myofascial trigger points (MTrPs) in the teres major muscle. Active MTrPs are hyperirritable spots in a palpable taut band that cause spontaneous pain, referred pain, restricted ROM and muscle weakness [17]. The pain described by Travell and Simons for active MTrPs in the teres major muscle is similar to the shoulder pain described by HB athletes [18].

Dry needling (DN) is an invasive technique that has shown grade A evidence of the treatment of MTrPs in the upper quarter [19]. This treatment consists of the introduction of a needle into the MTrPs [20]. DN has been shown to be effective in reducing pain intensity for nontraumatic shoulder pain [21,22]. However, the effects of DN in the teres major muscle has not been investigated in HB athletes with shoulder pain.

The aim of the current study was to determine the immediate effects of DN therapy in the active MTrPs of the teres major muscle in HB athletes with shoulder pain in pain intensity, glenohumeral ROM, isometric strength and extensibility of the tissues of the posterior part of the shoulder.

## 2. Materials and Methods

### 2.1. Study Design and Ethical Approval

A randomised single-blind controlled clinical trial was carried out between February and April 2021. The study design followed the Consolidated Standards of Reporting Trials (CONSORT) Guidelines. The study was registered at www.clinicaltrials.gov (accessed on 25 February 2021) and obtained the identification number NCT04777578. The Clinical Research Ethics Committee of Valladolid Este approved the study (CASVE-NM-21-504). The participants provided written and signed informed consent to participate in the study.

### 2.2. Sample Size

The sample size was calculated based on the primary outcome (pain intensity) using the Minitab 13.0 program. A value of 2.17 in the Numeric Pain Rating Score (NPRS) has been reported as the minimum clinically important difference (MCID) for shoulder pain [23] for the between-groups mean value. The standard deviation was based on a pilot study with HB athletes. Assuming a between-group mean value of 2.17 points and a standard deviation of 2.43 and estimating a two-tail test a level of significance of 0.05 and a follow-up loss rate of 15%, 30 participants were necessary for both groups (15 participants per group).

### 2.3. Participants

Thirty male patients (mean age 22.39 ± 3.73 years) were recruited from elite professional HB clubs in Soria (Spain).

The inclusion criteria were: male with unilateral shoulder pain and reproducible during throwing actions, 18–30 years of age, a minimum of 2 years’ experience practicing HB, practice routine of a minimum of 2 h/d and 3 d/w, a GIRD value ≥ 15° [12] and presence of an active MTrP in the teres major muscle. The identification of MTrP was performed using manual palpation. Manual palpation is the current criterion standard and has been shown to be reliable for the identification of MTrPs in the upper limb muscles [24]. The Travell and Simons criteria were used to identify the presence of MTrPs [18]: (1) presence of a palpable taut band; (2) appearance of local and/or referred pain when pressing the nodule of the taut band; and (3) recognition of the pain by the patient.

The exclusion criteria were: previous fracture, dislocation or surgery in any joint of the upper limb, cervical spine or thoracic spine, use of analgesics, muscle relaxants or other pharmacological treatment, previous physiotherapy treatment in the last month and DN contraindications (e.g., fear of needles).

### 2.4. Randomization and Masking

Thirty HB athletes were randomly allocated to both groups: the DN group or the control group. An independent assistant allocated the participants to the groups (ratio 1:1) using the GraphPad computer software 2018 (GraphPad Software, San Diego, CA, USA).

### 2.5. Interventions

DN intervention was performed by an experienced physical therapist with more than 7 years of clinical experience in the treatment of MTrPs with DN therapy. The intervention was ultrasound-guided to ensure the treatment of the targeted muscle. The therapist who applied the ultrasound had more than 10 years of experience in musculoskeletal sonography. Both therapists were blinded to all the measurements.

Participants allocated to the DN group received a single session of DN guided by ultrasound (SmartUS EXT-1M, REV:C; TELEMED, Lithuania, Vilnius, Lithuania) into active MTrPs in the teres major muscle. The active MTrP in the teres major muscle, that reproduced the symptoms of the patient, was treated. The ultrasound-guided technique provided a precise needle location within the teres major muscle, increasing the efficacy of treatment and minimizing risks. The transducer was first placed from the inferior angle of the scapula following the direction of the teres major muscle. The patient was lying in a prone position, and a 0.30 mm × 50 mm single-use stainless needle was inserted through the skin beside the ultrasound transducer using a guide tube [18].

Hong’s fast-in fast-out technique [25,26] was performed with the aim of eliciting the local twitch response. The quick contraction of the local muscle fibers in response to mechanical stimulation was detected by ultrasound visualization. The needle was repeatedly inserted until the local twitch responses became extinct [27]. A cotton ball was used to perform haemostasis and prevent bleeding after removing the needle.

No additional intervention was applied to the participants assigned to the control group. The participants remained in prone position for the same time that the intervention lasted in the DN group.

### 2.6. Outcomes

Outcome variables were measured at baseline and immediately after the intervention by two blinded examiners. Sociodemographic and clinical data were registered prior to testing. Pain intensity was the primary outcome, and passive ROM, isometric strength and extensibility of the posterior part of the shoulder were the secondary outcomes.

#### 2.6.1. Pain Intensity

Pain intensity during throwing action was recorded using the 11-point NPRS, in which 0 represented “no pain” and 10 “the most intense pain imaginable”. The reliability of the NPRS is good (Intraclass Correlation Coefficient (ICC)_2,1_ = 0.71–0.88) [28].

#### 2.6.2. Passive Range of Motion

The passive internal and external rotation ROM were recorded using a digital inclinometer following the procedure described by Fieseler et al. [29]. The patient was placed in a supine position with the shoulder at 90° of abduction and a towel under the arm to ensure the correct alignment of the upper limb in the frontal plane. The digital inclinometer was placed on the ventral part of the forearm, and the scapula was stabilized manually by the examiner. The internal and external rotation ROM was registered when the scapula was felt to move. The reliability of this protocol has been shown to be good for internal and external rotation (internal rotation: ICC_2,1_ = 0.79 (0.52–0.91); external rotation: ICC_2,1_ = 0.76 (0.48–0.89) [30]. In a preliminary intratester reliability, the ICC values obtained for these measurements were 0.88 for internal rotation and 0.90 for external rotation. Both shoulder joints (TS and non-TS) were measured.

After the passive ROM measurements, the GIRD and the ERG were calculated. The GIRD is considered the difference in internal rotation between the TS and the non-TS, described in negative value. The ERG is considered the difference in external rotation between both shoulder joints, described in positive value [29,30].

#### 2.6.3. Muscle Strength

The maximum isometric strength was measured following the procedure described by Romero-Franco et al. [31] using a hand-held dynamometer Lafayette model 01165. The patient was placed in the same position as described in the passive ROM measurement. The hand-held dynamometer was placed on the ventral part of the forearm or on the dorsal part of the forearm to assess internal rotation or external rotation isometric strength, respectively. The reliability of this protocol has been shown to be excellent for internal and external rotation (internal rotation: ICC_2,1_ = 0.99 (0.97–0.99); external rotation: ICC_2,1_ = 0.99 (0.98–0.99) [31]. Three trials were performed, and the maximum isometric force was taken. The ICC values achieved in a preliminary intratester reliability study were 0.96 for both measurements.

#### 2.6.4. Extensibility

The extensibility of the tissues of the posterior part of the shoulder was assessed using a digital inclinometer, according to the protocol described by Tyler et al. [32,33]. The patient was placed in side-lying position with the shoulder at 90° of abduction. The examiner manually stabilized the scapula. The angle was registered situating the digital inclinometer on the distal part of the humerus. If the humerus was horizontal, it was considered 0°; if below (adducted), it was recorded as a positive number; if above the horizontal (abducted), it was recorded as a negative number. This protocol has shown an excellent reliability (ICC)_2,1_ = 0.92) [32,33]. The ICC value obtained in a preliminary intratester reliability study was 0.95.

Primary and secondary outcome variables were measured before training sessions or warm-up.

### 2.7. Statistical Analysis

We performed statistical analyses using SPSS, version 20.0 for Windows. A *p*-value < 0.05 was considered statistically significant. Descriptive statistics were calculated to describe the sample. The Shapiro-Wilk test was used to calculate the normal distribution of the variables. The student’s t-test or the Mann-Whitney U test were used to compare demographic and clinical variables at baseline between both groups, according to the normally distributed data or non-normally distributed data, respectively. The chi-squared test or Fisher exact test were used to compare categorical data depending on the normality or non-normality distribution.

The Group by Time interaction between both groups (DN group and Control group) and time points (baseline and postintervention) were calculated using a two-way analysis of variance (ANOVA). The effect size (Cohen’s d) was also calculated to estimate the magnitudes of the within-group differences. The magnitude of the difference was classified as small if the value of Cohen’s d ranged from 0.2 to 0.5; as moderate if it ranged from 0.5 to 0.8; and as large if Cohen’s d was greater than 0.8. Moderate and large magnitudes of effect size were considered indicators of appropriate statistical power [34].

## 3. Results

**Figure 1:**
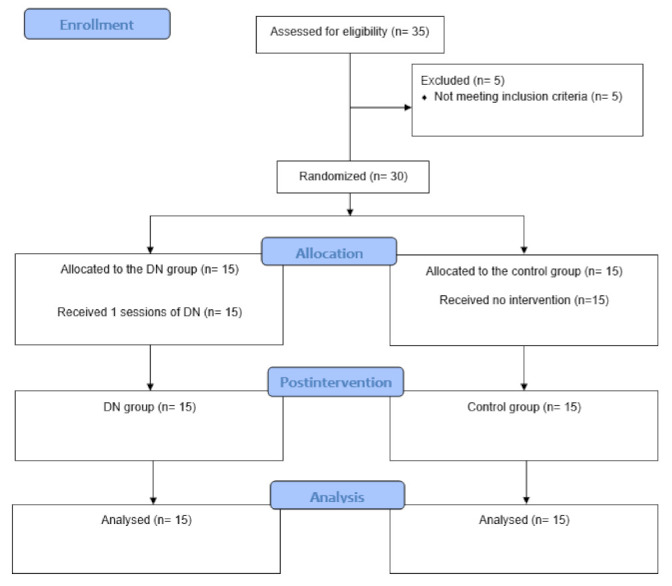
Flowchart diagram.

Between February and April 2020, 35 HB athletes with shoulder pain were screened for eligibility. Five HB athletes were not included for having received surgical interventions or physiotherapy treatment in the last month. No HB athletes were excluded for not presenting an MTrP in the teres major muscle. Finally, thirty HB athletes met all the eligibility criteria and were randomised to the DN group (*n* = 15) or to the control group (*n* = 15). The flowchart of the study is presented in Figure 1.

Sociodemographic and clinical characteristics of the HB athletes included are reported in Table 1. No statistically significant differences were found at baseline between both groups for any of the sociodemographic or clinical variables (*p* > 0.05).

A two-way ANOVA showed a significant Group by Time interaction after the intervention for pain intensity (F = 21.72; *p* < 0.001), internal rotation ROM (F = 102.70; *p* < 0.001), external rotation ROM (F = 5.57; *p =* 0.025), GIRD (F = 80.50; *p <* 0.001), ERG (F = 5.57; *p* = 0.025) and extensibility (F = 53.32; *p <* 0.001). The DN group presented higher changes than the control group in pain intensity (Δ 2.40 (0.92 to 3.88), internal rotation ROM (Δ −18.86 (−23.40 to −14.31), external rotation ROM (Δ 10.05 (3.36 to −16.74), GIRD (Δ −23.72 (−30.25 to −17.18), ERG (Δ 4.60 (−4.79 to 13.99) and extensibility (Δ −15.16 (−10.03 to −11.29). No between-groups or within-groups differences were found for maximum isometric strength (*p >* 0.05). Table 2 provides before and after treatment session data, within-group and between-groups differences and effect sizes.

## 4. Discussion

To the best of our knowledge, this is the first study to investigate the short-term effects of DN in the teres major muscle in HB athletes with shoulder pain. This randomised controlled trial showed that a single session of DN in the teres major muscle decreased pain during throwing actions, improved internal rotation ROM, GIRD and the extensibility of the tissues of the posterior part of the shoulder.

Pain intensity during the throwing action decreased after DN treatment. The change achieved in the DN group was higher than the MCID stated for patients with shoulder pain (2.17) [23]. These results are in agreement with previous studies that reported that one to three sessions of DN therapy in the infraspinatus muscle and upper trapezius muscle decreased pain intensity in the short-term in patients with shoulder pain [21,35]. However, this is the first study that investigated the effects of a single session of DN in the teres major muscle.

The analgesic effects may be related to the introduction of the needle into the dysfunctional motor endplate of the MTrP. DN therapy enhances the secretion of endogenous opioids, producing an immediate drop of proinflammatory cytokines and interleukins, neurotransmitters and neuromodulators [36,37].

Concerning shoulder ROM, Fieseler et al. [29] and Almeida et al. [12] showed that HB athletes with shoulder pain presented a GIRD greater than 15°. The HB athletes with shoulder pain included in this study presented a mean GIRD of −25.54° ± 8.38° (DN group: −25.63° ± 8.9°; Control group: −25.46° ± 8.13°). Therefore, the results reported in the current study at baseline are in agreement with previous studies that investigated the risk factors in HB athletes [12,29].

After the DN treatment, the internal rotation ROM increased, and the GIRD decreased. The mean values achieved on internal rotation ROM and GIRD were similar to the values reported for HB athletes without shoulder pain [12,29]. Burkhart et al. [7,10] described that the GIRD and the ERG could be explained because the restriction of the capsule and the ligament of the shoulder change the center of rotation of the humeral head to posterosuperior. However, the results achieved in this study showed that the teres major could play an additional role with the capsule and ligament structures, and its treatment may reverse these glenohumeral adaptations.

The extensibility of the tissues of the posterior part of the shoulder improved after the intervention in the DN group. The restriction of the tissues of the posterior part of the shoulder seems to be related to the changes in glenohumeral ROM [7,10]. Manske et al. [15] showed that the stretching of the tissues of the posterior part of the shoulder improved the internal rotation ROM. Despite this fact, few studies in the literature assessed the extensibility in different sports that involve throwing actions. However, the extensibility assessment could be an interesting variable to consider among throwing athletes.

The improvements achieved in the extensibility test may be related to the introduction of the needle into the MTrP. The needle provokes a disruption in the muscle fibers and motor endplates among other structures. The destruction of the dysfunctional motor endplates and the shortened sarcomeres of the taut band may explain these results [20,38].

Isometric maximum strength showed no between-groups or within-groups changes after the intervention. A recent meta-analysis suggested that DN has no effect on force production in patients with shoulder pain [39].

From a clinical viewpoint, the results reported in this randomised controlled trial showed that shoulder pain, internal rotation ROM, GIRD and the extensibility of the tissues of the posterior part of the shoulder in HB athletes seem to be linked to the presence of MTrPs in the teres major muscle. The intervention based on one single session of DN reported a statistically significant and clinically relevant decrease in pain intensity during throwing actions and an increase of internal rotation ROM, GIRD and extensibility of the tissues of the posterior part of the shoulder. According to these results, the treatment of the teres major muscle in HB athletes with shoulder pain may change pain intensity and reduce the primary risk factor for several injuries.

Several limitations need to be considered. First, only male HB athletes were included; thus, the results cannot be generalized to female patients or other athletes. Second, just one session was applied, and the results were measured immediately after the intervention, so no medium- or long-term effects were evaluated. Third, the outcomes were achieved after DN therapy; however, the management of shoulder pain should be multidisciplinary. Finally, future studies should investigate the combination of DN with other techniques in the short-, medium- and long-term in different types of athletes with shoulder pain.

## 5. Conclusions

This study showed that pain during the throwing action decreased, glenohumeral internal rotation ROM increased and extensibility improved after a single session of DN in active MTrPs of the teres major muscle in HB athletes with shoulder pain.

## Figures and Tables

**Table 1:** Sociodemographic and clinical outcomes.

Outcomes	DN Group	Control Group	*p*-Value
Age (years)	22.47 (3.04)	22.31 (4.37)	U = 106.50; *p* = 0.591
Height (cm)	182.20 (9.21)	186.25 (6.99)	F = 1.91; *p* = 0.177
Weight (Kg)	80.33 (10.80)	85.56 (11.52)	F = 1.69; *p* = 0.203
BMI (Kg/cm^2^)	24.17 (2.57)	24.64 (2.80)	F = 0.22; *p* = 0.637
NPRS	3.96 (2.20)	3.56 (2.84)	U = 101.00; *p* = 0.894
IR ROM (°)	22.00 (6.49)	25.78 (5.65)	F = 3.00; *p* = 0.109
ER ROM (°)	95.60 (10.54)	98.03 (6.21)	F = 0.62; *p* = 0.437
GIRD (°)	−25.63 (8.9)	−25.46 (8.13)	F = 0.01; *p* = 0.958
ERG (°)	8.43 (7.68)	5.40 (12.42)	F = 0.65; *p* = 0.425
IR strength (Kg)	13.06 (1.96)	13.93 (2.99)	F = 0.88; *p* = 0.353
ER strength (Kg)	16.52 (3.98)	16.25 (3.58)	F = 0.03; *p* = 0.844
Extensibility (°)	−14.43 (6.73)	−13.34 (4.76)	F = 0.27; *p* = 0.605

DN: Dry Needling; NPRS: Numeric Pain Rating Score; IR: Internal Rotation; ER: External Rotation; ROM: Range of Motion; GIRD: Glenohumeral Internal Rotation Deficit; ERG: External Rotation Gain.

**Table 2:** Baseline and postintervention outcomes as well as within group changes.

Outcomes	Baseline Mean (SD)	Postintervention Mean (SD)	Within-Group Changes	Effect Size	Between-Groups *p*-Values	Effect Size
Pain intensity						
DN group	3.96 (2.20)	0.65 (0.71)	−3.30 (2.02, 4.59) *p <* 0.001	2.0	F = 21.72; *p <* 0.001	1.3
Control group	3.56 (2.84)	3.06 (2.50)	−0.50 (−0,05, 1.05) *p =* 0.072	0.1		
IR ROM (°)						
DN group	22.00 (6.49)	45.26 (6.54)	23.26 (−28.12, −18.40) *p <* 0.001	3.5	F = 102.70; *p <* 0.001	3.0
Control group	25.78 (5.65)	26.40 (5.82)	0.62 (−1.53, 0.28) *p =* 0.164	0.1		
ER ROM (°)						
DN group	95.60 (10.54)	89.06 (11.07)	−6.53 (−0.41, 13.48) *p =* 0.063	0.6	F = 5.57; *p =* 0.025	1.0
Control group	98.03 (6.21)	99.12 (6.75)	1.09 (−2.69, 0.50) *p =* 0.166	0.1		
GIRD (°)						
DN group	−25.63 (8.9)	−2.50 (9.03)	23.13 (−27.96, −18.30) *p* < 0.001	2.5	F = 80.50; *p* < 0.001	2.6
Control group	−25.46 (8.13)	−26.21 (8.75)	−0.75 (−2.40, 3.90) *p =* 0.619	0.1		
ERG (°)						
DN group	8.43 (7.68)	1.90 (13.11)	−6.53 (−0.41, 13.48) *p =* 0.063	0.6	F = 5.57; *p =* 0.025	0.3
Control group	5.40 (12.42)	6.50 (12.44)	1.09 (−2.69, 0.50) *p =* 0.166	0.1		
IR Strength (Kg)						
DN group	13.06 (1.96)	13.51 (2.69)	0.44 (−1.53, 0.63) *p =* 0.392	0.1	F = 0.11; *p =* 0.773	0.2
Control group	13.93 (2.99)	14.18 (2.80)	0.25 (−0.78, 0.27) *p =* 0.321	0.1		
ER Strength (Kg)						
DN group	16.52 (3.98)	17.27 (4.35)	0.75 (−1.85, 0.35) *p =* 0.165	0.1	F = 0.02; *p =* 0.884	0.1
Control group	16.25 (3.58)	16.88 (4.34)	0.63 (−1.99, 0.73) *p =* 0.339	0.1		
Extensibility (°)						
DN group	−14.43 (6.73)	−0.8 (3.78)	13.63 (−17.19, −10.07) *p <* 0.001	2.5	F = 53.32; *p <* 0.001	2.9
Control group	−13.34 (4.76)	−15.96 (6.34)	−2.62 (−0.55, 5.80) *p =* 0.099	0.4		

SD: Standard Deviation; DN: Dry Needling; NPRS: Numeric Pain Rating Score; IR: Internal Rotation; ER: External Rotation; ROM: Range of Motion; GIRD: Glenohumeral Internal Rotation Deficit; ERG: External Rotation Gain.
